# Biobleaching application of cellulase poor and alkali stable xylanase from *Bacillus pumilus* SV-85S

**DOI:** 10.1007/s13205-012-0096-y

**Published:** 2012-10-10

**Authors:** Sushil Nagar, R. K. Jain, Vasanta Vadde Thakur, Vijay Kumar Gupta

**Affiliations:** 1 Department of Biochemistry, Kurukshetra University, Kurukshetra, 136119 India; 2 Biotechnology and Lignin By-products, Central Pulp and Paper Research Institute, Saharanpur, 247001 India

**Keywords:** Xylanase, Biobleaching, *Bacillus pumilus*, Solid state fermentation

## Abstract

The potential of extracellular alkali stable and thermo tolerant xylanase produced by *Bacilluspumilus* SV-85S through solid state fermentation was investigated in pulp bleaching in association with conventional bleaching using chlorine and chlorine dioxide. The biobleaching of kraft pulp with xylanase was the most effective at an enzyme dose of 10 IU/g oven dried pulp, pH 9.0 and 120 min incubation at 55 °C. Under the optimized conditions, xylanase pretreatment reduced Kappa number by 1.6 points and increased brightness by 1.9 points. Subsequently, chlorine dioxide and alkaline bleaching sequences (CDE_1_D_1_D_2_) finally resulted in brightness gain of 2.7 points as compared with the control. The pretreatment of pulp with xylanase resulted in 29.16 % reduction in chlorine consumption by maintaining the same brightness as in control. An improvement in pulp strength properties was also observed after bleaching of xylanase pretreated pulp. Scanning electron microscopy revealed loosening and swelling of pulp fibers after enzyme treatment. These results clearly demonstrated that the *B. pumilus* SV-85S xylanase was effective as a pulp biobleaching agent. The decrease in chlorine consumption by pretreatment of pulp with xylanase apparently made the biobleaching process not only economical but also eco-friendly.

## Introduction

The secondary cell walls of plants contain a wide range of additional compounds that modify their mechanical properties and permeability. The wood is composed of cellulose (35–50 %), xylan (20–35 %), and lignin (10–25 %). Lignin is a complex phenolic polymer that penetrates the spaces in the cell wall between cellulose, hemicellulose, and pectin components, driving out water and strengthening the wall. Lignin is present in all woody plants and other agro-residues which are used as raw materials for paper manufacturing. The removal of lignin is essentially required during paper manufacturing. The brown color of the pulp is due to the presence of lignin which has to be removed during paper making process (Viikari et al. 1986; Koponen 1991).

The pulp and paper industry are modifying its pulping, bleaching, and effluent treatment technologies to reduce the environmental impact of mill effluents. Pre-bleaching of kraft pulp with xylanase minimizes the chlorine required for bleaching, which in turn reduces chloro-organic discharges (Koponen 1991; Viikari et al. 1994). The process of delignification of pulp using enzymes was first presented by Viikari et al. (1994). Xylanase (EC 3.2.1.8), a hydrolytic enzyme, is being used primarily for the removal of the lignin–carbohydrate complex that is generated in the kraft process and acts as physical barrier during chemical bleaching. For biobleaching, xylanase must be stable and active at both high temperature and alkaline conditions (Srinivasan and Rele 1999). *Bacillus* strains are particularly attractive producers of high levels of extracellular cellulase-free xylanases stable at both high temperature and alkaline pH (Battan et al. 2007; Dhiman et al. 2008; Gupta et al. 2008; Nagar et al. 2011).

In our laboratory, a novel xylanolytic bacterial strain viz. *Bacillus pumilus* SV-85S has been isolated from soil. It produces high levels of alkali stable and cellulase poor xylanase, of which production has been optimized under both submerged fermentation (Nagar et al. 2010) and solid state fermentation (Nagar et al. 2011). The aim of this study was to investigate the potential application of this alkali stable xylanase as a biobleaching agent in pulp and paper industry.

## Materials and methods

### Microbial strain and its growth conditions

*Bacillus pumilus* SV-85S (MTCC 9861), our own isolate, is being maintained on nutrient agar (in g/L: peptone 5.0; beef extract 3.0 and agar 20.0) at 4 °C by transferring on to a fresh medium after every 4–6 weeks. The morphological, physiological, and biochemical characteristics of the isolated bacterial strain are already published (Nagar et al. 2010).

### Xylanase production and extraction

The inoculum was prepared by inoculating the autoclaved (at 121 °C and 1.05 kg/cm^2^ for 25 min) nutrient broth containing 0.5 % peptone and 0.3 % beef extract with a loop full of the overnight grown culture of *B. pumilus* SV-85S followed by incubation at 37 °C at 200 rpm. Xylanase was produced in Erlenmeyer flasks each containing 10 g of wheat bran and 30 mL mineral salt solution (in g/L: MgSO_4_·7H_2_O, 0.5; K_2_HPO_4_, 1.5; pH 8.0). The flasks were autoclaved at 121 °C (1.05 kg/cm^2^) for 25 min, cooled, inoculated with 15 % (v/w) of 18-h old inoculum and incubated at 30 °C. The flasks were gently tapped intermittently to mix the contents. The contents of the flasks were harvested after 48 h of incubation. All the production experiments were conducted in triplicates and standard deviation (SD) was calculated to show the variability among the replicated flasks. The detailed optimized production of xylanase by *B. pumilus* SV-85S has already been reported (Nagar et al. 2011). Xylanase was extracted from the fermented carbon source with 100 mL of distilled water by squeezing through a muslin cloth followed by centrifugation at 10,000×*g* for 30 min at 4 °C. The clear supernatant (crude extract) was used for enzyme assay and biobleaching studies.

### Enzyme assay

The xylanase activity was assayed according to the method of Bailey et al. (1992) by measuring the amount of reducing sugars (xylose equivalent) liberated from xylan using 3,5-dinitrosalicylic acid (Miller 1959). The reaction mixture containing 490 μL of 1 % birch wood xylan (Sigma) as substrate, and 10 μL of appropriately diluted enzyme extract was incubated at 50 °C for 5 min. The reaction was then terminated by adding 1.5 mL of 3,5-dinitrosalicylic acid reagent (in g/L: 3,5-dinitro-2-hydroxybenzoic acid, 10.0; sodium potassium tartrate, 200.0; sodium hydroxide, 10.0; sodium sulfite, 0.5; phenol, 2.0). A control was run simultaneously that contained all the reagents, but the reaction was terminated prior to the addition of enzyme. The contents were placed in a boiling water bath for 10 min followed by cooling in ice-cold water. The absorbance of the resulting color was measured against the control at 540 nm in a spectrophotometer.

Cellulase activity (CMCase, i.e., carboxymethyl cellulase and FPase, i.e., filter paper digesting activity) was determined as described in Nagar et al. (2011). One unit (IU) of xylanase or cellulase activity was defined as the amount of enzyme that catalyzed the release of 1 μmol of reducing sugar as xylose or glucose equivalent per minute under the specified assay conditions.

### Biobleaching of kraft pulp (E_0_CDE_1_D_1_D_2_)

Xylanase produced by *B. pumilus* SV-85S under optimized submerged fermentation conditions was used for pre-bleaching of kraft pulp so as to evaluate its potential use as biobleaching agent.

The unbleached kraft pulp composed of different types of wood (eucalyptus, bamboo, poplar, eucalyptus rulla, vaneer, debarka bamboo hardwood, etc.) was cooked at 180 °C for 60 min at a pressure of 8.0–10.0 kg/m^3^ and dried in an oven at 42–48 ± 2 °C till a constant weight was attained. The consequential pulp was further used for biobleaching application. The optimum pretreatment conditions were decided by measuring Kappa number, reducing sugars, brightness, yellowness, and whiteness of the control, and enzyme-treated pulp samples. The experiment was laid down in completely randomized block design with different doses of the enzyme, temperature, retention time, and pH as individual treatments with three replications. The data were analyzed using Statistical Analysis Software (SAS) package and PROC GLM. The mean of different treatment effects were compared using least significant difference (LSD).

### Enzyme pretreatment of kraft pulp (E_0_)

The oven dried pulp at 10 % (w/v) consistency was pretreated with *B. pumilus* SV-85S xylanase at a dose of 10 IU/g pulp in polyethylene bags under optimized conditions at pH 9.0 and incubated at 55 °C for 120 min in a water bath. An untreated pulp sample (as control) was also incubated simultaneously under the identical conditions. The control and xylanase pretreated pulps were thoroughly washed and dewatered by a 75-mesh screen and then subjected to conventional chemical bleaching sequence (CDE_1_D_1_D_2_). A part of the control and xylanase-treated pulps were used to prepare hand sheets according to Technical Association of Pulp and Paper Industry (TAPPI) standard methods so as to evaluate their brightness. The pulp samples were also analyzed for Kappa number.

### Chlorine, chlorine dioxide treatment (CD)

The xylanase pretreated kraft pulp at 3 % consistency was subjected to treatment with a mixture of chlorine:chlorine dioxide (in the ratio of 90:10) keeping total chlorine constant at 4.14 % at pH 2.0 for 1 h at 50 °C. The pulp was then filtered and washed with distilled water. The resulting filtrate was analyzed to determine the amount of chlorine consumed. A part of the washed pulp was made into hand sheets to determine its brightness (according to TAPPI). The remaining pulp was used for the next bleaching step.

### Alkali treatment (E_1_) step

The washed kraft pulp (at 3 % consistency) obtained after treatment with chlorine:chlorine dioxide was treated with sodium hydroxide (2.5 %) at 60 °C for 2 h. The alkali was removed by thorough washing with distilled water. A part of the washed pulp was made into hand sheets to determine its brightness. The remaining pulp was used for the next bleaching step.

### Chlorine dioxide step (D_1_D_2_)

The pulp (collected after alkali step) at 10 % consistency was treated sequentially with 0.9 and 0.1 % chlorine dioxide to remove traces of lignin at a temperature of 70 °C. The pulp was filtered and washed to determine the amount of chlorine dioxide consumed during the treatment. The washed pulps were made into hand sheets to determine their brightness, yellowness, and whiteness. The strength properties such as tensile strength, breaking length, burst factor, burstness, tear factor, and tearness pulp of the hand sheets were also determined.

### Analysis of pulp and paper properties

The hand sheets prepared from untreated and xylanase-treated pulps were evaluated for various physical properties following the TAPPI protocols (TAPPI Test Methods, Atlanta, GA, TAPPI Press, 1996). All the measurements were conducted in triplicate, with their averages and SD calculated. The Kappa number (a measure of lignin content), was determined by the treating the pulp samples with acidified potassium permanganate (TAPPI Protocol, T-236 om-85). The brightness (T 452 om-98), yellowness, and whiteness of the hand sheets were measured at 457 nm with ISO Colourtech (USA). The strength properties viz. tensile strength (T 231 om-96), breaking length (T 404 cm-92), burstness, burst factor (T 403 om-97), tear factor, and tearness (T 414 om-98) were tested according to standard methods of TAPPI. The amount of total chlorine in the biobleached effluents was also determined to calculate the chlorine consumed during the bleaching process.

### Scanning electron microscopy (SEM)

The surface of xylanase treated and an untreated kraft pulp sample was observed with SEM. The samples were dehydrated in an ascending grade of acetone, critical point dried (Critical point dryer, Polaron), and mounted on aluminium stubs. They were sputter coated (SCD 050 Super Cool Sputter System; Baltec Technology, Liechtenstein) with colloidal gold and observed under a Leo 435 VP scanning electron microscope (Cambridge, UK) at an operating voltage 15 kV. Images were digitally acquired by using a CCD camera attached to the microscope. The magnification for observing the loosening and swelling of pulp fibers was 350× and 1500×, respectively.

## Results and discussion

### Application of xylanase in biobleaching of kraft pulp

Under optimized solid state fermentation conditions, *B. pumilus* SV-85S produced 73000 IU/g (~7300 IU/mL) of xylanase (Nagar et al. 2011). Further, cellulase activity was negligible (CMCase 0.017 IU/g and FPase 0.026 IU/g) in the cell-free supernatant, indicating that xylanase was more or less cellulase free. The xylanase was found to be completely stable over a broad pH (5–11) range and retained 52 % of its activity upon incubation at 70 °C for 30 min (Nagar et al. 2011). Its molecular weight was 23.7 kDa which was lower as compared with several other xylanases. The molecular weight of this xylanase was determined by gel filtration chromatography through Sepharose 6B as well as by SDS-PAGE. These characteristics of *B. pumilus* SV-85S xylanase together with its economical production using wheat bran (a cost-effective substrate) suggested its potential implication as a biobleaching agent.

The application of *B. pumilus* SV-85S xylanase in biobleaching of kraft pulp was investigated according to E_0_CDE_1_D_1_D_2_ sequence in which the E_0_ stage refers to enzymatic pre-bleaching and CDE_1_D_1_D_2_ indicates conventional chemical bleaching. So, the pulp was pretreated with xylanase followed by bleaching with conventional chemical sequence CDE_1_D_1_D_2_. The role of xylanase in reducing chlorine consumption maintaining the same brightness as in control was also investigated. The use of xylanase in pre-bleaching has been given special attention as it may reduce the consumption of chlorine compounds by up to 30 %, so that a 15–20 % reduction in organochlorines in the effluents could be achieved (Polizeli et al. 2005). The utilization of xylanases could lead to replacement of 5–7 kg chlorine dioxide per ton of kraft pulp and an average decrease of 2–4 U in the Kappa number, a measure of lignin content in the cellulose pulp (Polizeli et al. 2005).

### Enzymatic pretreatment of pulp (E_0_)

#### Parametric optimization for pulp pretreatment with xylanase

The process conditions viz. enzyme dose, incubation temperature, retention time, and pH were optimized for pre-bleaching of oven dried kraft pulp with xylanase so as to obtain effective dispersion of the enzyme. During optimization, various observations such as Kappa number, reducing sugars, brightness (%ISO), yellowness, and whiteness were recorded. These parameters formed the basis for determining the efficiency of enzyme pretreatment. A decrease in Kappa number and increase in brightness would indicate a better efficiency of the enzyme treatment.

The effect of varying enzyme dose on the efficiency of pretreatment was investigated at pH 7.0, temperature 50 °C, and incubation time of 120 min. The data in Table 1 revealed that the least mean Kappa number (18.3) was found at enzyme dose of 10 IU/gds, followed by 12.5 and 15.0 IU/gds (18.4). The next higher value of Kappa number was at 7.5 IU/gds (18.5). However, all these values are statistically at par with each other. The maximum Kappa number was found in control. The measurement of brightness at different enzyme doses revealed that the brightness was maximum (36.7 %ISO) in control and at the enzyme dose of 10 IU/gds followed by the values at 12.5 IU (36.5), 15 IU (36.4), and 7.5 IU (35.7). However, all these values of brightness at 7.5–15.0 IU/gds enzyme were statistically at par. The lowest brightness was found at 5.0 IU/gds. Though statistically at par with 7.5 IU, 10 IU/gds has been selected as the optimum enzyme dose for further experiments because of the lowest mean Kappa number and highest brightness at this dose. An enzyme dose of 10 IU/g pulp was found to be optimum for biobleaching of mixed hardwood kraft pulp by several researchers (Sindhu et al. 2006; Ahlawat et al. 2007; Dhiman et al. 2009; Sanghi et al. 2009; Garg et al. 2011). In contrast, maximum bleaching was reported with enzyme dose more than 10 IU/g (Kulkarni and Rao 1996; Bissoon et al. 2002; Khandeparkar and Bhosle 2007).

**Table 1:** Parametric optimization for pretreatment of kraft pulp with xylanase by *B. pumilus* SV-85S

Enzyme dose (IU/gds)	Kappa no.	Reducing sugars (g/50 g pulp)	Brightness %ISO	Yellowness	Whiteness
Optimization of enzyme dose					
Control	19.1^c^	0.00^b^	36.7^a^	15.6^b^	−28.6
5.0	18.8^bc^	0.173^a^	35.1^b^	15.4^ab^	−27.9
7.5	18.5^ab^	0.180^a^	35.7^a^	15.3^a^	−27.8
10.0	18.3^a^	0.190^a^	36.7^a^	15.2^a^	−27.2
12.5	18.4^a^	0.188^a^	36.5^a^	15.2^a^	−27.0
15.0	18.4^a^	0.187^a^	36.4^a^	15.2^a^	−27.0
CD (*p* = 0.05)	0.28	0.07	0.95	0.27	

Temperature (°C)	Kappa no.	Reducing sugars (g/50 g pulp)	Brightness %ISO	Yellowness	Whiteness
Optimization of treatment temperature					
50	18.3^c^	0.190	36.1^b^	15.2	−27.2
55	17.8^a^	0.202	35.7^c^	15.3	−26.9
60	18.1^bc^	0.193	36.7^a^	15.2	−27.1
65	18.0^ab^	0.191	36.5^a^	15.2	−27.1
70	18.0^ab^	0.190	36.4^ab^	15.2	−27.0
CD (*p* = 0.05)	0.24	NS	0.32	NS	

Retention time (min)	Kappa no.	Reducing sugars (g/50 g pulp)	Brightness %ISO	Yellowness	Whiteness
Optimization of retention time					
0	18.7	0.185	36.0	15.8^c^	−27.3
60	18.5	0.187	35.9	15.6^b^	−27.1
120	18.1	0.190	36.1	15.2^a^	−27.1
180	18.3	0.188	35.8	15.2^a^	−27.0
CD (*p* = 0.05)	NS	NS	NS	0.26	

pH	Kappa no.	Reducing sugars (g/50 g pulp)	Brightness %ISO	Yellowness	Whiteness
Optimization of pH					
7	18.2^c^	0.190	36.1^b^	15.2	−27.1
8	18.1^c^	0.196	36.4^ab^	15.1	−27.2
9	17.1^a^	0.216	36.7^a^	14.8	−27.1
10	17.6^b^	0.202	36.6^a^	14.9	−27.1
CD (*p* = 0.05)	0.20	NS	0.40	NS	

In a column, different letters indicate statistically significant differences between the means (*p* < 0.05)

All the experiments were performed with 10 IU of xylanase at pH 7.0, temperature 50 °C, and incubation period of 120 min, except the variable parameter

*CD* critical difference, *NS* not significant

The effect of varying temperature on the efficiency of pretreatment was investigated at an enzyme dose 10 IU/g, pH 7.0, and incubation time 120 min. A temperature of 55 °C was found to be the most effective for pretreatment of kraft pulp by *B. pumilus* SV-85S xylanase as it resulted in the lowest Kappa number (Table 1). However, it was statistically at par with the Kappa number at 65 and 70 °C. The maximum Kappa number was observed at 50 °C. The measurement of brightness at different temperatures revealed that the brightness was maximum (36.7 %ISO) at 60 °C, which was statistically at par with that at 65 and 70 °C. Changes in reducing sugars at different temperatures were statistically non-significant. Since Kappa number was the lowest at 55 °C, this temperature was used for further experiments. Several researchers have reported optimum temperature in the range of 50–60 °C for pulp biobleaching (Beg et al. 2000; Sindhu et al. 2006; Battan et al. 2007; Dhiman et al. 2009; Garg et al. 2011).

The effect of varying retention time on the efficiency of pretreatment was investigated at an enzyme dose 10 IU/g, pH 7.0, and temperature 55 °C. The maximum efficiency of the enzyme in biobleaching was obtained after 120 min of incubation which caused reduction in mean Kappa number from 18.7 to 18.2, though it was statistically non-significant as compared with the values at 0, 60, and 180 min (Table 1). The longer periods of incubation did not enhance the extent of biobleaching benefits significantly. So, in subsequent experiments, enzyme was incubated with the pulp for 120 min. A similar incubation time (2 h) has been reported by various workers (Beg et al. 2000; Khandeparkar and Bhosle 2007; Sanghi et al. 2008; Ko et al. 2011). However, several researchers showed an optimum incubation time of 3 h (Garg et al. 1998; Bim and Franco 2000; Dhillon and Khanna 2000; Bissoon et al. 2002; Sindhu et al. 2006; Kiddinamoorthy et al. 2008).

The effect of pH on the efficiency of pretreatment was studied at an enzyme dose 10 IU/g pulp, retention time 120 min, and temperature 50 °C. The biobleaching efficiency of *B. pumilus* SV-85S xylanase was found to be the best at pH 9.0 as it showed the lowest Kappa number and maximum brightness (Table 1). At other pH values, Kappa number was higher and brightness was less than the values at pH 9.0. The release of reducing sugars was statistically non-significant at different pH values. The optimum working efficiency of xylanase used in this study at pH 9.0 was in accordance with earlier reports (Garg et al. 2011; Kaur et al. 2011). However, the majority of cited work documented the optimum pH in the range of 6.0–8.0 (Chandralata et al. 2004; Sanghi et al. 2008; Manimaran et al. 2009; Ko et al. 2011).

The results on the optimization of enzymatic pre-bleaching revealed that bleaching was the most effective at an enzyme dose 10 IU/g pulp upon incubation for 2 h at 55 °C and pH 9.0.

#### Pretreatment with xylanase under optimized conditions

Pretreatment of pulp with xylanase (E_0_ stage) under the above optimized conditions reduced the Kappa number by 1.6 points and increased the brightness from 36.9 ± 0.12 to 38.8 ± 0.20 %ISO (Table 2) indicating effectiveness of the enzyme in pulp bleaching. Several researchers have reported an increase in brightness and decline in Kappa number but with variable magnitude following the xylanase pretreatment (Jiang et al. 2006; Ahlawat et al. 2007; Sanghi et al. 2008; Garg et al. 2011). The increase in brightness after enzyme pretreatment might be due two reasons. First, the xylanase would act on the xylan precipitated on the lignin (Viikari et al. 1986). This xylan was precipitated due to lowering of the pH at the end of cooking stage. Its removal by the action of xylanase would enhance the accessibility of bleaching chemicals to the pulp fibers. The second reason was based on the ability of lignin to form complexes with polysaccharide such as xylan and some of the bonds being alkali-resistant might not have been hydrolyzed during the kraft process (Buchert et al. 1992). The xylanase acted by cleaving the remaining bonds between lignin and xylan, opening the structure of the cellulose pulp and leading to the fragmentation of xylan and subsequent extraction of the fragments (Paice et al. 1978). Treatment with xylanase rendered the pulp more permeable to subsequent chemical extraction of the residual brown lignin and lignin–carbohydrate from the fiber (Lei et al. 2008).

**Table 2:** Effect of *B. pumilus* SV-85S xylanase treatment on chlorine consumption

Parameters	Control (CDE_1_D_1_D_2_)	Enzyme treated (E_0_CDE_1_D_1_D_2_)				
		100 %	90 %	80 %	70 %	60 %
Enzyme treatment stage (E_0_)						
Pulp consistency (%)	10	10				
Enzyme dose (U/g)	Nil	10				
Temperature (°C)	55	55				
Retention time (min)	120	120				
pH	9.0	9.0				
Kappa no.	18.8 ± 0.17	17.2 ± 0.28				
Brightness ISO (%)	36.9 ± 0.12	38.8 ± 0.20				
Cl_2_–ClO_2_ treatment Stage (CD)						
Cl_2_ added (%)	4.14	4.14	3.73	3.31	2.90	2.48
Cl_2_ consumed (%)	4.08	3.91	3.46	3.23	2.86	2.41
Brightness ISO (%)	52.2 ± 2.32	56.9 ± 0.16	55.6 ± 1.09	55.5 ± 2.18	55.5 ± 1.22	53.5 ± 1.22
Alkali treatment stage (E_1_)						
Alkali added (%)	2.5	2.5	2.5	2.5	2.5	2.5
H_2_O_2_ added (%)	1.0	1.0	1.0	1.0	1.0	1.0
Brightness ISO (%)	57.2 ± 3.12	62.9 ± 1.12	61.6 ± 2.17	61.7 ± 0.11	61.8 ± 1.70	57.8 ± 2.20
ClO_2_ treatment stage (D_1_)						
ClO_2_ added (%)	0.80	0.80	0.72	0.64	0.56	0.48
ClO_2_ consumed (%)	0.72	0.7	0.69	0.62	0.54	0.46
Brightness ISO (%)	78.9 ± 22.2	81.9 ± 25.12	80.1 ± 13.07	79.7 ± 18.21	78.2 ± 11.20	77.1 ± 13.0
ClO_2_ treatment stage (D_2_)						
ClO_2_ added (%)	0.1	0.1	0.09	0.08	0.07	0.06
ClO_2_ consumed (%)	0.07	0.07	0.06	0.06	0.05	0.06
Brightness ISO (%)	81.2 ± 2.32	83.6 ± 1.37	82.4 ± 2.17	82.3 ± 1.91	81.3 ± 0.62	78.8 ± 1.42
Yellowness	1.71 ± 0.17	1.41 ± 0.18	1.48 ± 0.29	1.53 ± 0.80	1.89 ± 0.22	2.02 ± 0.19
Whiteness	78.0 ± 1.37	80.0 ± 2.18	82.4 ± 3.29	82.3 ± 2.90	80.1 ± 4.21	77.1 ± 3.42
Comparative results						
Total chlorine used	4.87	4.68	4.21	3.91	3.45	2.93
Chlorine saved (%)	0.00	3.90	13.55	19.71	29.16	39.84
Brightness ISO (%)	81.2	83.9	86.6	85.7	81.8	78.2
Unit improved (%)	0.00	2.96	1.48	1.35	0.12	−2.95

The results showed that xylanase may be employed as the first step for boosting pulp bleaching. The desirable properties of xylanase to be exploited for pulp bleaching include cellulase free/poor nature and active at both high temperature and alkaline pH. The cellulase free/poor nature of xylanase would preserve the cellulose fibers. All these characteristics are fulfilled by *B. pumilus* SV-85S xylanase used in this study for pulp bleaching (Nagar et al. 2010, 2011). Moreover, xylanase in crude form could be employed for pulp bleaching.

#### Chemical bleaching (CDE_1_D_1_D_2_)

The xylanase pretreated pulp when subjected to CDE_1_D_1_D_2_ bleaching sequence resulted in notable increase in brightness as compared with control after each stage of processing (Table 2). The enhancement in brightness in both the pulp samples was highest at D_1_ stage. The final gain in brightness of enzyme pretreated pulp (83.9 %ISO) was 2.7 points as compared with the control (81.2 %ISO) demonstrating efficacy of the enzymatic pre-bleaching. Li et al. (2005) reported that pretreatment with cellulase-free xylanase isolated from the fungus *Thermomyces lanuginosus* brightened the pulp by 1.8 %ISO over the control.

To evaluate the potential of xylanase from *B. pumilus* SV-85S in reducing the chlorine consumption, chemical bleaching of enzyme pretreated pulp was carried out by successively reducing the amount of chlorine to obtain the same brightness as in the control. It was evident from the data shown in Table 2 that pretreatment with the *B. pumilus* SV-85S xylanase finally resulted in 29.16 % reduction in chlorine consumption by maintaining the same brightness level as in control. The decrease in chlorine consumption by the pretreatment of pulp with xylanase apparently made the biobleaching process not only economical but also eco-friendly. These results clearly showed that the *B. pumilus* SV-85S xylanase was effective as pulp biobleaching agent and it was more efficient as compared to several other xylanases reported earlier. Enzymatic biobleaching with xylanase from *B. pumilus* ASH resulted in 20 % reduction in chlorine consumption without any change in brightness (Battan et al. 2007). Xylanase from *B. stearothermophilus* SDX reduced the chlorine consumption up to 15 % while its combination with pectinase resulted in 20 % reduction (Dhiman et al. 2008). Treatment of eucalyptus pulp with commercial xylanases such as Novozyme 473, and Cartazyme HS-10 reduced the chlorine consumption by 31 % and increased the final brightness by 2.1–4.9 points (Bajpai et al. 1994). Xylanase P (a commercial enzyme) improved the brightness of kraft pulp by 5.6 points when used at 10 U/g of moisture free pulp and caused 10 % reduction in chlorine consumption (Madlala et al. 2001). The greater bleaching potential of *B. pumilus* SV-85S xylanase used in the present investigation might be due to its lower molecular mass, stability at alkaline pH and higher temperatures and negligible cellulase activity.

#### Effect of xylanase treatment on paper quality

The pulp subjected to E_0_CDE_1_D_1_D_2_ bleaching sequence showed improvement in its physical properties as compared with the control. The data given in Table 3 revealed a gain in various strength properties such as tensile strength, breaking length, burst factor, burst, tear factor, and tearness in handmade paper sheets of enzyme-treated pulp compared wih untreated pulp. Thus, enzyme treatment resulted in an improvement in the quality of paper. The favorable effect of xylanase treatment of paper strength properties of pulp has been documented by various workers (Ninawe et al. 2006; Battan et al. 2007; Garg et al. 2011; Kaur et al. 2011; Ko et al. 2011). Dhiman et al. (2008) reported significant improvement in various physical properties through single and mixed lay out strategy. Beg et al. (2000) also recorded an improvement in tensile strength and burst factor by up to 63 and 8 %, respectively, by xylanase produced from *Streptomyces* sp. QG-11-3. Xylanase from *Staphylococcus* sp. SG-13 has been shown to increase the tensile strength and burst factor by 10 and 17 % respectively (Gupta et al. 2000).

**Table 3:** Effect of *B. pumilus* SV-85S xylanase treatment on properties of bleached pulp

Pulp properties	Control	Enzyme treated	Gain (%)
Tensile strength (N)	57.3 ± 1.56	67.2 ± 2.96	17.28
Breaking length (m)	6830 ± 8.23	7413 ± 9.34	8.69
Burst factor	38.1 ± 1.18	40.9 ± 1.28	7.34
Burstness (lb/in.^2^)	43.24 ± 4.56	45.88 ± 5.66	6.10
Tear Factor	61.6 ± 2.31	68.0 ± 3.87	10.38
Tearness (mN)	192 ± 12.34	210 ± 13.45	10.52

#### SEM of xylanase-treated pulp

**Fig. 1:**
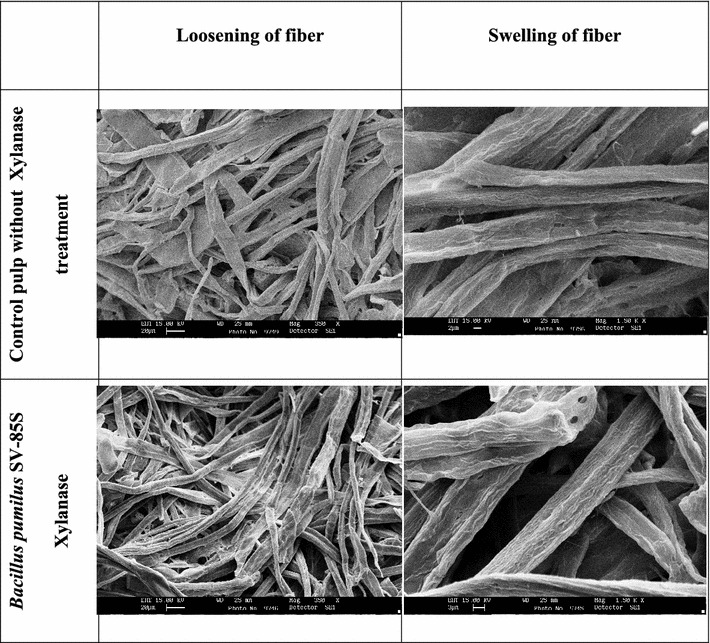
SEM micrographs of untreated and xylanase-treated paper pulp showing loosening and swelling of fibers

SEM studies clearly showed morphological changes in the pulp fibers after treatment with crude xylanase. A comparison of SEM images from the control and SV-85S xylanase-treated samples revealed an increase in loosening and swelling of pulp fibers after treatment with the enzyme (Fig. 1). The surface of untreated pulp fibers appeared smooth, whereas that of the treated fibers was rough. These changes in pulp fibers resulted in its refining facilitating enhanced accessibility of chemicals used in subsequent bleaching stages. The fiber swelling also improved physical properties of the pulp. Similar disruption and separation of pulp fibers upon enzyme treatment by SEM was reported earlier (Poorna and Prema 2007; Sanghi et al. 2009).

## Conclusion

Exploiting xylanase for pre-bleaching has been given special attention in view of the environmental regulations that have restricted the use of chlorine compounds in bleaching processes in the paper and cellulose industries. The present investigation revealed that enzymatic pretreatment of pulp with cellulase-free, thermo tolerant, and alkali stable xylanase produced by *B. pumilus* SV-85S reduced the chlorine consumption by 29.16 % with the same brightness level as in control. This enzyme is apparently superior to the most other available xylanases, which have been shown to reduce chlorine consumption up to 20 %. Pre-bleaching with xylanase also led to improvement in various pulp strength properties like tensile strength, breaking length, burst factor, burstness, tear factor, and tearness. The above characteristics of *B. pumilus* SV-85S enzyme together with its economical production using cost-effective and abundantly available wheat bran in high levels would facilitate its exploitation as biobleaching agent in pulp and paper industry leading to substantial reduction in chlorine consumption thereby minimizing the risk of environmental pollution.
